# Aptamer Molecular Beacon Sensor for Rapid and Sensitive Detection of Ochratoxin A

**DOI:** 10.3390/molecules27238267

**Published:** 2022-11-26

**Authors:** Hao Yu, Qiang Zhao

**Affiliations:** 1 State Key Laboratory of Environmental Chemistry and Ecotoxicology, Research Center for Eco-Environmental Sciences, Chinese Academy of Sciences, Beijing 100085, China; 2 University of Chinese Academy of Sciences, Beijing 100049, China; 3 School of Environment, Hangzhou Institute for Advanced Study, UCAS, Hangzhou 310024, China

**Keywords:** ochratoxin a, aptamer, fluorescence, molecular beacon, sensor

## Abstract

Ochratoxin A (OTA) is a carcinogenic fungal secondary metabolite which causes wide contamination in a variety of food stuffs and environments and has a high risk to human health. Developing a rapid and sensitive method for OTA detection is highly demanded in food safety, environment monitoring, and quality control. Here, we report a simple molecular aptamer beacon (MAB) sensor for rapid OTA detection. The anti-OTA aptamer has a fluorescein (FAM) labeled at the 5′ end and a black hole quencher (BHQ1) labeled at the 3′ end. The specific binding of OTA induced a conformational transition of the aptamer from a random coil to a duplex–quadruplex structure, which brought FAM and BHQ1 into spatial proximity causing fluorescence quenching. Under the optimized conditions, this aptamer sensor enabled OTA detection in a wide dynamic concentration range from 3.9 nM to 500 nM, and the detection limit was about 3.9 nM OTA. This method was selective for OTA detection and allowed to detect OTA spiked in diluted liquor and corn flour extraction samples, showing the capability for OTA analysis in practical applications.

## 1. Introduction

Ochratoxin A (OTA) is a common toxin and carcinogenic fungal secondary metabolite produced by *Aspergillus ochraceus*, *Penicillium verrucosum*, and *Penicillium nordicum* [1]. Many food stuffs such as nuts, beer, wine, grapes, processed animal feedstock, and cereals, etc., can be contaminated with OTA throughout the world. Ingestion of OTA-contaminated foods can cause serious toxicological effects to humans, including mutagenic, teratogenic, hepatotoxic, immunotoxic, and neurotoxic effects [2,3]. Considering the wide distribution and high toxicity of OTA, the Agency for Research on Cancer (IARC) has classified OTA as a potential human carcinogen (group 2B) [4]. The European Union has set the permissible level of OTA in food at 2 to 5 ng/mL (5.0–12.4 nM). Therefore, it is of great significance to detect OTA for food safety and environmental monitoring. Diverse strategies have been developed for OTA detection, such as mass spectrometry (MS) [5], high performance liquid chromatography (HPLC) [6], thin layer chromatography (TLC) [7], and so on. These methods have some major shortcomings, including elevated cost, experienced operators, complex procedures, and expensive instruments. Thus, there is an urgent need to develop a simple and rapid method for OTA detection.

Aptamers are single-stranded DNA or RNA molecules with selective binding characteristics and high binding affinity to targets, which were first reported in 1990 by an in vitro procedure termed Systematic Evolution of Ligands by Exponential Enrichment (SELEX) [8,9]. Compared with antibodies, aptamers offer remarkable advantages such as small batch-to-batch variation, high thermal stability, ease of chemical synthesis, and easy modification of functional moieties [10,11,12]. Since the DNA aptamer against OTA was successfully selected [13], various aptamer-based signal transduction assays have been reported, such as fluorescence assays [14,15], colorimetric assays [16,17], electrochemical assays, and etc., and some of them need aptamer immobilization, separation or tedious steps for analysis [18]. It is crucial to develop a simple and sensitive assay for OTA with high speed.

Molecular aptamer beacon (MAB) techniques are widely used in the fields of biotechnology, chemistry, medical sciences, biochip construction, and biosensor development due to their unique characteristics such as simplicity, sensitivity, high speed, and the mechanism of fluorescent signal transduction [19,20,21]. The assays rely on target binding-induced aptamer structure switching and subsequent change in fluorescence intensity. The fluorescent molecular aptamer beacon assays were applied to detect a variety of targets including proteins (e.g., thrombin, Tat-1 protein) and small molecules (e.g., metal ions, adenosine triphosphate, nicotinamide adenine dinucleotide), showing the merits of simplicity and sensitivity [21,22,23,24,25].

In this work, taking advantage of aptamer and molecular beacon strategy, we developed a simple and rapid fluorescence method for OTA detection by using a high-affinity DNA aptamer against OTA, which has a duplex and G-quadruplex structure in the affinity complex [26]. The aptamer was modified with a fluorescein (FAM) and a black hole quencher (BHQ1) at the 5′ terminal and the 3′ terminal, respectively. In the presence of OTA, the aptamer underwent a large conformation change, drawing FAM and BHQ1 into close proximity, and the fluorescence of FAM was quenched by BHQ1. By testing a series of aptamers with different lengths of stems, the 33 mer aptamer probe with a short four-base paired stem, denoted as OT-Bea-4bp, was used as a fluorescent probe, which exhibited the largest fluorescence change upon OTA binding. We optimized some conditions for OTA detection with the proposed method, including CaCl_2_ concentration and NaCl concentration in binding buffer, buffer pH, and incubation time. Under the optimal experimental conditions, we achieved sensitive and selective detection of OTA with short time by using the probe OT-Bea-4bp, and as low as 3.9 nM OTA could be detected. The aptamer beacon-based fluorescence method also allowed to detect OTA in complex sample matrixes. Compared with other strategies, our method only requires aptamer probes labeled with FAM and BHQ1 at both terminal ends and one simple mixing-measuring step, without the need for aptamer immobilization, using nanomaterials for signal amplifications, and tedious separation and detection steps. This aptamer molecular beacon sensor is promising in simple, rapid, and sensitive detection of OTA in applications although this sensor shows turn-off response to target binding.

## 2. Results and Discussion

### 2.1. Principle of Molecular Aptamer Beacon Sensor for OTA

**Figure 1:**
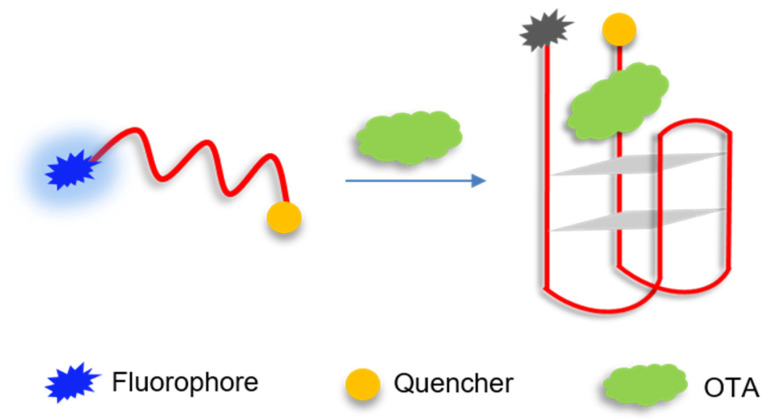
Principle of molecular aptamer beacon sensor for OTA.

Figure 1 illustrates the principle of molecular aptamer beacon sensor for OTA. The aptamer against OTA has a fluorescein (FAM) labeled at the 5′ end and a black hole quencher 1 (BHQ1) labeled at the 3′ end. In the absence of OTA, the FAM is distant from the BHQ1, resulting in high fluorescence intensity. In the presence of OTA, OTA specifically binds to the aptamer, inducing the aptamer folding into a duplex–quadruplex structure [26], and the fluorescence is quenched due to the close proximity of FAM and BHQ1. Therefore, OTA detection can be achieved by measuring the change in fluorescence intensity of the aptamer probe upon OTA binding.

**Figure 2:**
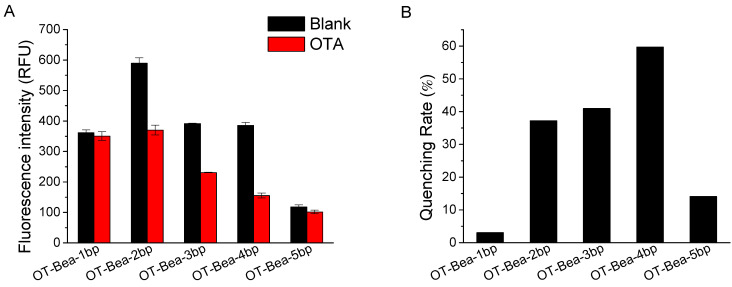
(**A**) Fluorescence intensity of aptamer probes (50 nM) with different lengths of stem in the absence of and in the presence of OTA (500 nM). (**B**) Quenching rate ((1 − F_OTA_/F_Blank_) × 100%, F_OTA_ and F_Blank_ represent the fluorescence intensity of OTA sample and blank sample, respectively) of aptamers having different stem lengths from 1 bp to 5 bp. Binding buffer was 20 mM Tris-HCl (pH 7.5), 120 mM NaCl, and 20 mM CaCl_2_.

Aptamer undergoes a large conformation change upon OTA binding, which is important for signal generation in MAB sensor and facilitates sensitive detection of OTA [22]. We designed aptamers with different numbers of base pairs in the stem ranging from 1 bp to 5 bp by truncating the aptamer against OTA [13], and tested the fluorescence signal responses of FAM-labeled aptamers in the absence of and in the presence of OTA (Figure 2). The fluorescence intensity of the tested aptamer probes decreased when OTA existed, suggesting OTA binding induced aptamer structure change, which brought FAM closer to BHQ1. OT-Bea-1bp showed negligible fluorescence change in the presence of OTA as a result of instability of the stem structure, which had weak affinity to OTA. OT-Bea-2bp, OT-Bea-3bp, and OT-Bea-4bp exhibited significant fluorescence quenching in the presence of OTA, suggesting that OTA binding caused a conformational change in aptamer and brought FAM and BHQ1 into close proximity. OT-Bea-5bp exhibited lower fluorescence intensity in the absence of OTA, and OTA binding induced a small fluorescence signal change. OT-Bea-2bp showed high fluorescence in the absence of OTA as it had an unstable stem and open conformation. With the aptamer stem lengths increased from 2 bp to 5 bp, the fluorescence intensity of the aptamer probes gradually decreased in the absence of OTA. The results indicate that the aptamer with longer stem may already have a stable stem structure when OTA is absent and the FAM and the BHQ1 are adjacent to each other. In addition, the nucleotide (e.g., G) adjacent to fluorophore can cause some fluorescence quenching of the fluorophore labeled on DNA [14,27], so the fluorescence of aptamer probes can also be affected by the nucleotides in the stem. It is expected that OT-Bea-3bp shows higher fluorescence than OT-Bea-4bp in the absence of OTA. However, OT-Bea-3bp provided similar fluorescence to OT-Bea-4bp, which is possibly because the FAM at OT-Bea-3bp is close to G at the terminal and a fluorescence quenching caused by the adjacent G occurs at OT-Bea-3bp. For OT-Bea-1bp, the observed lower fluorescence in the absence of OTA may be due to that the stem was too short and FAM was close to BHQ1. Furthermore, the FAM at the 5′ end of OT-Bea-1bp was close to the multiple Gs and the fluorescence of FAM was partly quenched by the adjacent multiple Gs [14,27]. Among the tested aptamer probes, upon OTA binding, OT-Bea-4bp showed the largest fluorescence quenching rate ((1 − F_OTA_/F_Blank_) × 100%) with the value of nearly 60%, while the quenching rates of OT-Bea-2bp and OT-Bea-3bp were 37% and 41%, respectively. Therefore, the probe OT-Bea-4bp was chosen for construction of the MAB sensor for OTA as it showed higher fluorescence change upon OTA binding.

### 2.2. Optimization of Experimental Conditions

To obtain the best sensing performance for OTA detection, we optimized some experimental conditions, including CaCl_2_ concentration in binding buffer, NaCl concentration in binding buffer, buffer pH, and incubation time.

**Figure 3:**
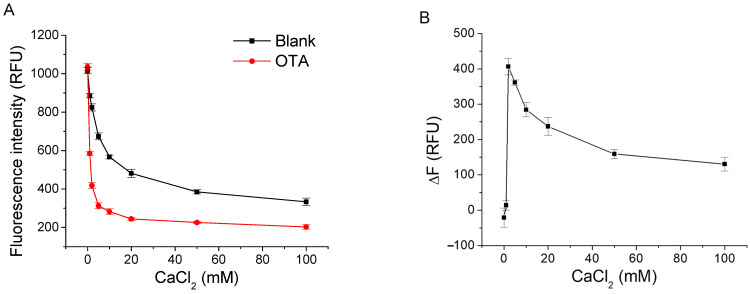
(**A**) Effect of CaCl_2_ in buffer on the fluorescence intensity of 50 nM OT-Bea-4bp in the absence and presence of 200 nM OTA. (**B**) Effect of CaCl_2_ in the buffer on the fluorescence intensity change (ΔF, F_Blank_ − F_OTA_) induced by OTA. Binding buffer was 20 mM Tris-HCl (pH 7.5), 120 mM NaCl, 0.1% Tween 20, and various concentrations of CaCl_2_.

We tested the effect of CaCl_2_ on the fluorescence intensity response of OT-Bea-4bp with and without OTA in the solution containing 20 mM Tris-HCl (pH 7.5), 120 mM NaCl, and 0.1% Tween 20. As shown in Figure 3, with the addition of CaCl_2_ up to 50 mM, the fluorescence intensity of the blank sample and the sample containing OTA dramatically decreased, and then slightly changed when CaCl_2_ concentration was higher than 50 mM. With the increase in CaCl_2_ from 0 to 2 mM, the fluorescence signal change (ΔF, F_Blank_-F_OTA_) induced by OTA sharply increased to a maximum value of about 400, and then slowly decreased with the increasing in CaCl_2_ concentration. The results indicate that CaCl_2_ concentration is a critical factor for the aptamer affinity [14]. We chose 2 mM CaCl_2_ for the subsequent experiments because large signal change was induced by OTA at this condition.

NaCl concentration in the binding buffer also exhibited great influence on the detection of OTA by OT-Bea-4bp (Figure S1 in Supporting Information). The fluorescence intensity of OT-Bea-4bp increased with increasing in NaCl concentration for the samples with and without OTA. A larger fluorescence signal change (ΔF) caused by OTA binding was observed when buffer contained 120 mM NaCl, and further increasing in NaCl was unfavorable for the generation of large signal change upon OTA addition. Therefore, 120 mM NaCl was applied to the subsequent experiments.

We further investigated the influence of buffer pH on the responses of the MAB sensor (Figure S2 in Supporting Information). With the buffer pH increased from 5.5 to 8.5, the fluorescence intensity of OT-Bea-4bp showed a remarkable increase, reached the maximum value when buffer pH was 8.5, and then remained unchanged as the pH increased from 8.5 to 10.5. Upon the addition of OTA, the fluorescence intensity change (ΔF) dramatically increased when the buffer pH increased from 5.5 to 8.5, and further increase in pH did not change the value of ΔF. Buffer pH at 8.5 was applied to detection of OTA.

These results show that the concentrations of CaCl_2_ and NaCl and the pH of practical samples can have large effects on the detection of OTA, so sample dilution with the binding buffer and other treatments are needed in the practice of the method to reduce these effects.

We also tested the effect of incubation time on OTA detection in the MAB assay with the optimum binding buffer of 20 mM Tris-HCl (pH 8.5), 2 mM CaCl_2_, 120 mM NaCl, and 0.1% Tween 20 (Figure S3 in Supporting Information). The aptamer probe OT-Bea-4bp immediately showed remarkable response to OTA once OTA was added. Longer incubation time resulted in slight changes in fluorescence intensity, and the signal changes caused by OTA were almost the same with different incubation time. Clearly, the MAB sensor was rapid in responding OTA. We chose an incubation time of 15 min for the detection of OTA. An incubation time shorter than 15 min can be applied for faster detection to meet the demand in higher speed.

### 2.3. Determination of OTA

**Figure 4:**
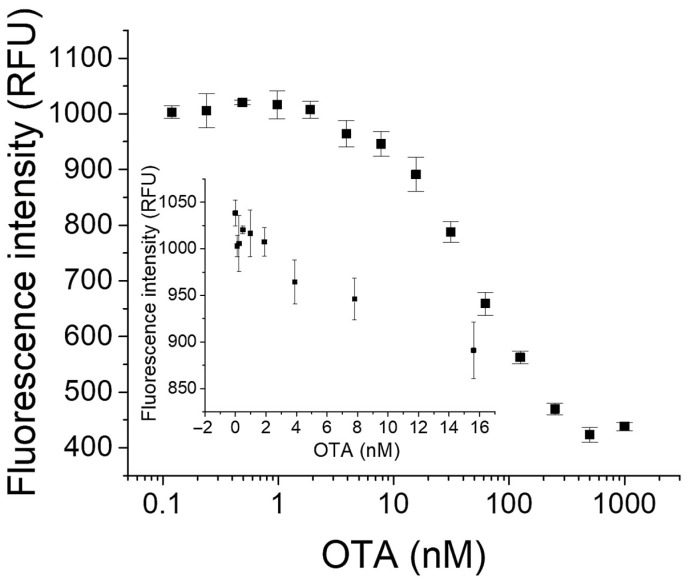
Detection of OTA with the aptamer probe OT-Bea-4bp. The inset shows the fluorescence responses of the aptamer probes to blank samples and low concentrations of OTA.

We successfully achieved detection of different concentrations of OTA under the optimal conditions by using the aptamer probe OT-Bea-4bp. As shown in Figure 4, the fluorescence intensity gradually decreased with the increasing in OTA concentration. OTA in the concentration range from 3.9 nM to 500 nM could be detected, and the detection limit was calculated to be about 3.9 nM (S/N = 3). Reducing the fluorescence fluctuations in measurement and errors in sample preparation may further improve the detection limit. The quenching rate of OT-Bea-4bp reached nearly 63% when 1 μM OTA existed. The dissociation constant (*K*_d_) of OT-Bea-4bp was estimated to be 16 ± 2 nM by non-linear fitting with GraphPad according to previous report [28]. The detection limit of this MAB sensor of OTA detection is lower than or comparable to some of the previously reported aptamer-based methods for OTA [14,15,16,17,29,30,31,32,33], and our MAB method shows the advantages in simplicity, speed, and sensitivity.

### 2.4. Selectivity Test

**Figure 5:**
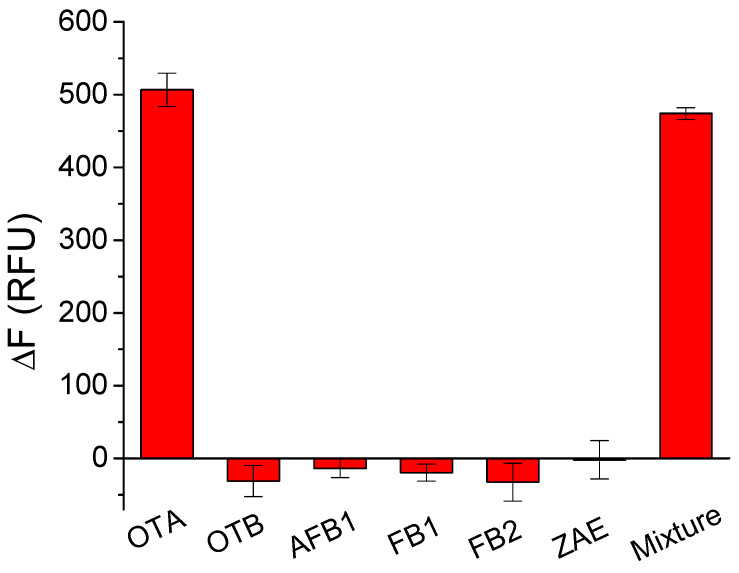
Selectivity test of the MAB assay for OTA using aptamer probe OT-Bea-4bp. The concentrations of all the tested mycotoxins were 200 nM. The mixture contained 200 nM OTA, OTB, AFB1, FB1, FB2, and ZAE.

To confirm the selectivity of the MAB sensor for OTA, we tested other mycotoxins including OTB, AFB1, FB1, FB2, and ZAE. As shown in Figure 5, the presence of OTA resulted in a significant decrease in fluorescence intensity, while the addition of other mycotoxins caused some fluorescence increase or little fluorescence change over that of the blank sample. The existence of other mycotoxins did not significantly interfere with the detection of OTA. The results indicate that the MAB sensor was selective for OTA.

### 2.5. OTA Detection in Complex Samples

To further assess the possible practical application of the proposed assay, we tested different concentrations of OTA spiked in 100-fold diluted corn flour extraction and 50-fold diluted liquor with binding buffer (Figure S4 in Supporting Information). The performances of OT-Bea-4bp exhibited almost the same fluorescence signal for OTA in the diluted complex sample matrix to that of binding buffer. The detection limits of OTA in the 100-fold diluted corn flour extraction and 50-fold diluted liquor were estimated to be 7.8 nM, which were slightly higher than that in the binding buffer. For the original samples, the detection limits would be higher. The results demonstrate that the MAB sensor has potential in practical application. To achieve the detection of low levels of OTA in real samples, sample pretreatment, and target preconcentration can be applied.

## 3. Experimental Section

### 3.1. Materials and Reagents

Ochratoxin A (OTA), ochratoxin B (OTB), fumonisin B1 (FB1), fumonisin B2 (FB2), aflatoxin B1 (AFB1), and zearalenone (ZAE) were purchased from Pribolab (Singapore). CaCl_2_, NaCl, and Tween 20 were obtained from Sinopharm. All DNA oligonucleotides having fluorescein (FAM) labeled at the 5′ end and black hole quencher 1 (BHQ1) labeled at the 3′ end were synthesized and purified with HPLC by Sangon Biotech (Shanghai, China). The sequences are list in Table 1. Samples of corn flour and liquor wine were purchased from a local supermarket. The black 96-well microplates were ordered from Thermo Fisher Scientific Inc. (USA). Ultrapure water was obtained from a system of Elga Labwater (Purelab Ultra Genetic type, UK). Other reagents were of analytical grade.

### 3.2. OTA Detection

Varying concentrations of OTA were mixed with a fixed concentration of aptamer probes (OT-Bea-4bp) in the same binding buffer of 20 mM Tris-HCl (pH 8.5), 120 mM NaCl, 2 mM CaCl_2_, and 0.1% Tween 20, and the final concentrations of OTA and OT-Bea-4bp were 0–1 μM and 50 nM, respectively. After incubation for 15 min at room temperature, 100 μL of the sample mixture was transferred into a black 96-well microplate, and fluorescence intensity was measured immediately with the excitation wavelength of 485 nm and the emission wavelength of 528 nm by a microplate reader (BioTek Synergy H1, USA). Each sample was measured three times and the average data were used.

### 3.3. OTA Detection in Complex Sample Matrixes

The liquor sample was first filtered through a 0.22 μm membrane, and then ultra-filtered through a Millipore filter at a centrifugation speed of 12,000 rpm for 15 min at 25 °C (Cut off: 3 KDa). Then, different concentrations of OTA were added to the 2% diluted liquor sample, and the fluorescence intensity was measured using OT-bea-4bp with the same assay procedure as described above. For OTA detection in diluted corn flour samples, 1 g corn flour sample was extracted with 3 mL methanol/water (7:3, *v*/*v*) mixture for 5 min with vigorous shaking, then centrifuged at 12,000 rpm for 20 min. The supernatant was first filtered with a 0.22 μm membrane and then ultra-filtered through a Millipore filter (Cut off: 3 KDa) at 12,000 rpm for 15 min. Finally, we tested different concentrations of OTA in 1% diluted corn flour samples.

## 4. Conclusions

In summary, we successfully developed a simple and rapid molecular aptamer beacon sensor for OTA. The rationally designed aptamer had a FAM labeled at the 5′ end and a BHQ1 labeled at the 3′ end. OTA binding induced the aptamer to undergo a significant conformational change, bringing FAM and BHQ1 into a close proximity, and caused a decrease in fluorescence intensity. Sensitive OTA detection was achieved by monitoring the fluorescence changes. This simple molecular aptamer beacon sensor enabled to detect OTA with a detection limit of 3.9 nM. This method was selective for OTA and allowed detection of OTA spiked in diluted complex sample matrixes, showing the potential in applications.

## Figures and Tables

**Table 1:** Sequences of DNA oligonucleotides.

Name	Sequence (5′ to 3′)
OT-Bea-1bp	FAM-TCG GGT GTG GGT GGC GTA AAG GGA GCA-BHQ1
OT-Bea-2bp	FAM-ATC GGG TGT GGG TGG CGT AAA GGG AGC AT-BHQ1
OT-Bea-3bp	FAM-GAT CGG GTG TGG GTG GCG TAA AGG GAG CAT C-BHQ1
OT-Bea-4bp	FAM-CGA TCG GGT GTG GGT GGC GTA AAG GGA GCA TCG-BHQ1
OT-Bea-5bp	FAM-GCG ATC GGG TGT GGG TGG CGT AAA GGG AGC ATC GC-BHQ1

Complementary nucleotides are shown in underlined format.

## Data Availability

Not applicable.

## Supplementary Materials

The following supporting information can be downloaded at: https://www.mdpi.com/article/10.3390/molecules27238267/s1, Figure S1: Effects of NaCl concentration in buffer on the fluorescence response of OT-Bea-4bp to OTA; Figure S2: Effects of buffer pH on the fluorescence response of OT-Bea-4bp to OTA; Figure S3: Effects of incubation time on the fluorescence response of OT-Bea-4bp to OTA; Figure S4: Detections of OTA spiked in 100-fold diluted corn flour extraction or 50-fold diluted liquor with OT-Bea-4bp; Table S1: Comparison of some aptamer-based methods for OTA.
