# Supplementary material for: Aptamer Molecular Beacon Sensor for Rapid and Sensitive Detection of Ochratoxin A
Source: Molecules. 2022 Nov 26;27(23):8267. doi: 10.3390/molecules27238267 (PMC9737911; doi:10.3390/molecules27238267)
Supplement: Supplementary file 1 [file molecules-27-08267-s001.zip › molecules-2029979-supplementary.pdf]

Supporting information

Aptamer Molecular Beacon Sensor for Rapid and Sensitive Detection of Ochratoxin A

Hao Yu [1,2] and Qiang Zhao*[1,2,3]

1. State Key Laboratory of Environmental Chemistry and Ecotoxicology, Research Center for Eco-Environmental Sciences, Chinese Academy of Sciences, Beijing, 100085, China

2. University of Chinese Academy of Sciences, Beijing, 100049, China

3. School of Environment, Hangzhou Institute for Advanced Study, UCAS, Hangzhou 310024, China

[*] Corresponding Author, Email address: qiangzhao@rcees.ac.cn

Table S1 Comparison of some aptamer-based methods for OTA

Strategy	Dynamic range	LOD	Analysis time	Ref.
A direct fluorescence anisotropy assay using tetramethylrhodamine-labeled aptamer.	3 nM-3 μM	3 nM	40 min	[14]
A competitive aptamer fluorescence anisotropy assay.	2.5-1250 nM	2.5 nM	15 min	[15]
Label-free aptasensor using SYBR Gold and exonuclease I.	20-500 nM	16.5 nM	60 min	[29]
A FRET aptasensor.	0-160 nM	14 nM	30 min	[30]
FRET aptasensor using CD served as energy donor and Ag nanoparticles as energy donor.	10 nM-5 μM	8.7 nM	<30 min	[31]
Aptamer-based colorimetric assay.	20-625 nM	20 nM	3 min	[16]
Aptasensor based on exonuclease-assisted recycling amplification.	5-200 nM	0.96 nM	40 min	[32]
Colorimetric aptasensor based on gold nanoparticle.	6.3-750 nM	5 nM	70 min	[17]
Aptamer fluorescent sensor based on photoinduced electron transfer between guanine and fluorophore.	3-300 nM	1.3 nM	30 min	[33]
Molecular aptamer beacon sensor	3.9 nM-500 nM	3.9 nM	15 min	This work

FRET: Forster resonance energy transfer, CD: nitrogen doped carbon dots.

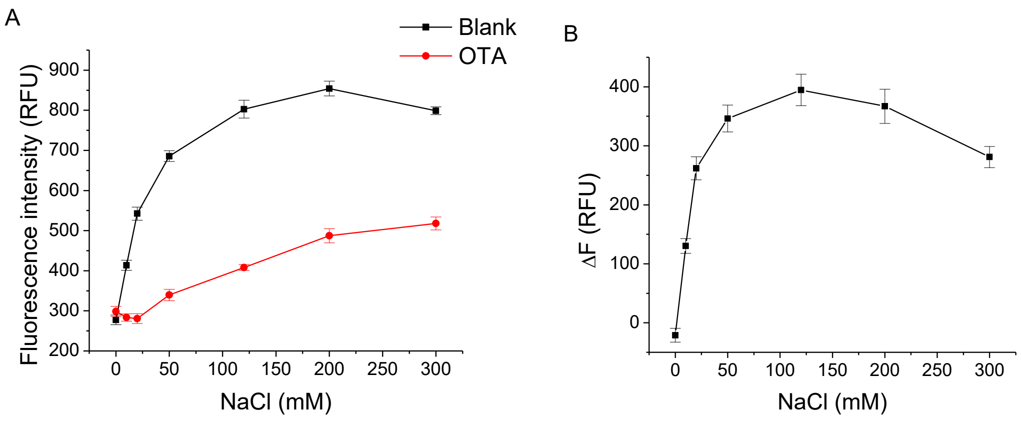

Figure S1. (A) Effects of NaCl concentration in buffer on the fluorescence response of OT-Bea-4bp (50 nM) in the absence and in the presence of 200 nM OTA. (B) Effects of NaCl concentration in the buffer on the fluorescence intensity change (ΔF, F_{Blank}-F_{OTA}) induced by OTA. Binding buffer contained 20 mM Tris-HCl (pH 7.5), 2 mM $CaCl_2$, 0.1% Tween 20, and various concentrations of NaCl.

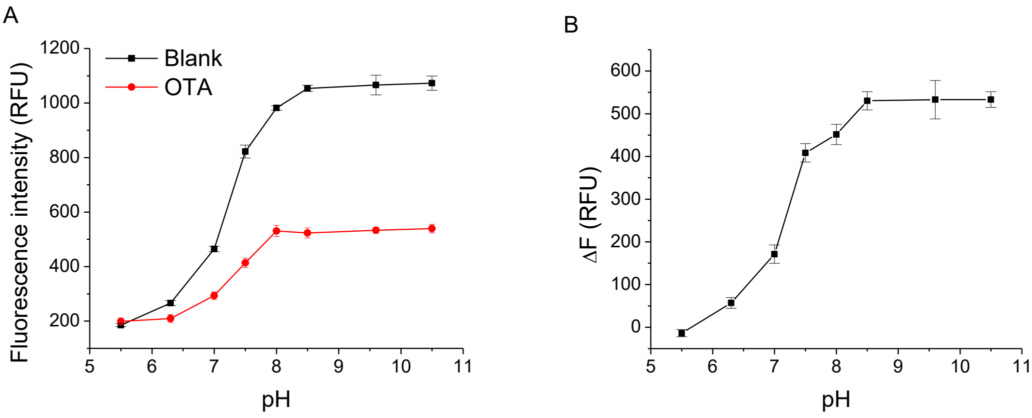

Figure S2. (A) Effects of buffer pH on the fluorescence response of 50 nM OT-Bea-4bp in the absence and in the presence of 200 nM OTA. (B) Effects of buffer pH on the fluorescence intensity change (ΔF, F_{Blank}-F_{OTA}) induced by OTA. Binding buffer was the solution containing 20 mM Tris-HCl, 2 mM $CaCl_2$, 120 mM NaCl, and 0.1% Tween 20.

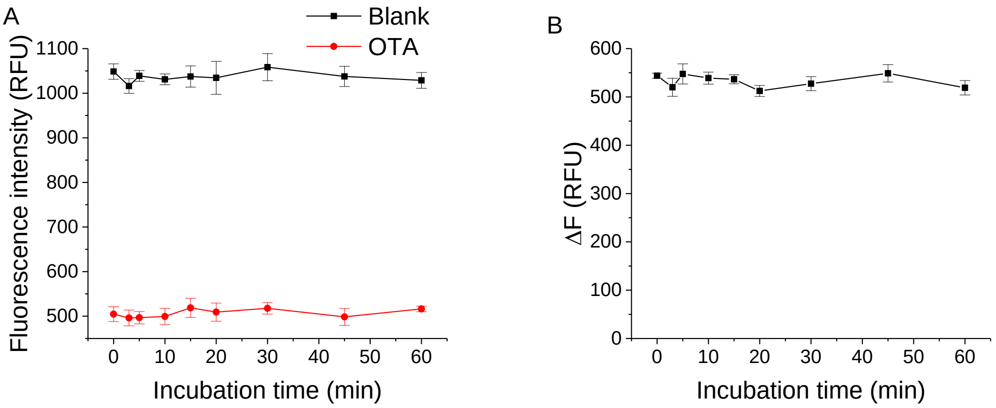

Figure S3. (A) Effects of incubation time on the fluorescence response of OT-Bea-4bp (50 nM) in the absence and in the presence of 200 nM OTA. (B) Effects of incubation time on the fluorescence intensity change (ΔF, F_{Blank}-F_{OTA}) induced by OTA. Binding buffer: 20 mM Tris-HCl (pH 8.5), 2 mM $CaCl_2$, 120 mM NaCl, and 0.1% Tween 20.

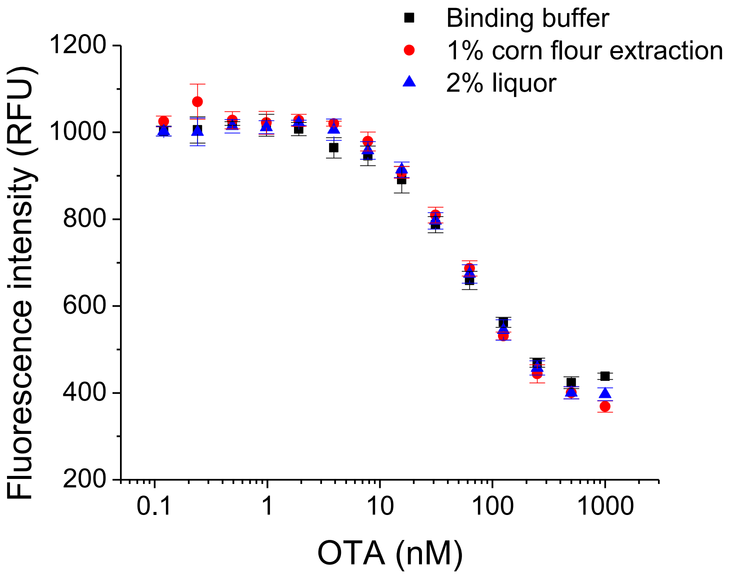

Figure S4. Detections of OTA spiked in 100-fold diluted corn flour extraction or 50-fold diluted liquor with OT-Bea-4bp.
